# Porcine coronavirus promotes NLRP3 inflammasome activation during virus-induced neuroinflammation

**DOI:** 10.1128/mbio.01725-26

**Published:** 2026-08-17

**Authors:** Qianyu Zhou, Junchao Shi, Ruizhao Qiu, Rui Dai, Yungang Lan, Jiyong Zhou, Xuming Deng, Wenqi He, Zi Li

**Affiliations:** 1 State Key Laboratory for Diagnosis and Treatment of Severe Zoonotic Infectious Diseases, Key Laboratory for Zoonosis Research of the Ministry of Education, Institute of Zoonosis, and College of Veterinary Medicine, Jilin University 12510, Changchun, Jilin Province, China; 2 Collaborative innovation center and State Key Laboratory for Diagnosis and Treatment of Infectious Diseases, First Affiliated Hospital, Zhejiang University 12377, Hangzhou, Zhejiang Province, China; 3 MOA Key Laboratory of Animal Virology, Center for Veterinary Sciences, Zhejiang University 12377, Hangzhou, Zhejiang Province, China; The University of Iowa, Iowa City, Iowa, USA

**Keywords:** coronavirus, neuroinflammation, porcine coronavirus, NLRP3, nucleocapsid protein

## Abstract

Neurotropic coronaviruses can invade the central nervous system (CNS) and cause severe neurological disease, but the mechanisms underlying virus-induced neuroinflammation remain incompletely understood. Here, we report that infection with porcine hemagglutinating encephalomyelitis virus (PHEV), a neurotropic betacoronavirus, is associated with mitochondrial dysfunction, including increased reactive oxygen species (ROS) production and loss of the mitochondrial membrane potential (ΔΨm). Our data identify the NOD-like receptor family pyrin domain-containing 3 (NLRP3) inflammasome as an important component connecting PHEV infection, mitochondrial stress, and PANoptosis. The viral nucleocapsid (N) protein interacted with the pyrin domain (PYD) of NLRP3 through its C-terminal region and facilitated inflammasome assembly without detectably disrupting the NLRP3-NIMA-related kinase 7 (NEK7) interaction. Pharmacological inhibition of NLRP3 or caspase-1, as well as genetic knockout of NLRP3, significantly reduced viral load, attenuated neuroinflammation, and delayed disease progression in PHEV-infected mice. Moreover, NLRP3 deficiency shifted the cell-death modality from PANoptosis to necroptosis, thereby mitigating CNS immunopathology. Together, these findings demonstrate that PHEV hijacks the NLRP3-mitochondrial axis to exacerbate neuroinflammatory injury and support further evaluation of inflammasome- and PANoptosis-related pathways as therapeutic targets for neurotropic coronavirus infections.

**IMPORTANCE:**

Porcine hemagglutinating encephalomyelitis virus (PHEV) is a neurotropic betacoronavirus that primarily invades the central nervous system (CNS) and induces fatal encephalomyelitis in young piglets. However, the host and viral determinants that contribute to PHEV-induced brain injury remain incompletely defined. In this paper, we show that PHEV infection activates the host NOD-like receptor family pyrin domain-containing 3 (NLRP3) inflammatory pathway and that this response contributes to CNS immunopathology. Loss of NLRP3 shifts the infection-associated cell death program from PANoptosis to necroptosis. Blocking this pathway reduces viral load, attenuates neuroinflammation, and improves survival in infected mice. These findings expand our understanding of host responses to neuroinvasive coronaviruses and highlight inflammasome signaling as a candidate therapeutic target for controlling coronavirus-associated encephalitis in swine.

## INTRODUCTION

Neurotropic RNA viruses pose a persistent threat to human and animal health and can cause severe neurological dysfunction through dysregulated neuroinflammatory responses (1–3). Inflammasomes are innate immune signaling platforms that have context-dependent roles in viral neuropathogenesis, contributing not only to antiviral defense but also to inflammatory tissue injury when dysregulated. These multiprotein complexes respond to pathogen-associated and damage-associated molecular patterns (PAMPs and DAMPs), homeostasis-altering molecular processes (HAMPs), and effector-triggered immunity (ETI), leading to inflammatory cytokine release and lytic cell death (4–6). Understanding the intricate interplay between neurotropic viruses and inflammasome activation is therefore paramount for developing therapeutic strategies against viral encephalitis and mitigating neurological sequelae.

Coronaviruses (CoVs) are a diverse family of RNA viruses with a broad host range, impacting both human and animal health (7, 8). Some neurotropic CoVs can invade the central nervous system (CNS), where they may cause neurological dysfunction and inflammatory injury. Although inflammasome activation has been implicated in the immunopathology of SARS-CoV-2 and mouse hepatitis virus (MHV) infection (9–11), its role in other pathogenic betacoronaviruses, particularly those with strong neurotropism, remains less well defined. The neurotropic betacoronavirus, Porcine hemagglutinating encephalomyelitis virus (PHEV), causes acute encephalomyelitis with robust neuroinflammation in young pigs and mice (12–16). Previous studies have shown that PHEV infection is associated with microglial activation, blood-brain barrier disruption, and enhanced neuropathology, raising the possibility that inflammasome signaling contributes to PHEV-induced CNS inflammation (15). However, whether and how PHEV engages inflammasome pathways to drive neuroinflammation are not yet understood.

Several distinct pattern recognition receptors (PRRs) nucleate inflammasomes, including NOD-like receptor thermal protein domain-associated protein 1 (NLRP1), CARD8, NLRP3, NLRP6, NLR family CARD domain-containing 4 (NLRC4), Absent in melanoma 2 (AIM2), pyrin, and non-canonical caspases (4, 17). Among these sensors, NLRP3 stands out as the most extensively characterized inflammasome-nucleating receptor, capable of integrating diverse danger signals, including pathogen infection, ion flux disturbances, organelle stress, and metabolic alterations (18–20). Structurally, NLRP3 contains the N-terminal pyrin domain (PYD), nucleotide-binding domains (NACHT), and leucine-rich repeat domains (LRR) (21, 22). Upon activation by sensing PAMPs/DAMPs, the NLRP3 PYD domain binds to the PYD domain of apoptosis-associated speck-like protein containing a CARD (ASC), initiating ASC oligomerization and subsequent CASP1 activation. This process culminates in caspase-1 (CASP1)-mediated maturation of interleukin-1β (IL-1β) and IL-18, alongside gasdermin D (GSDMD)-driven pyroptosis (23). Following cleavage, the GSDMD N-terminus (NT) oligomerizes to form plasma membrane pores that facilitate IL-1β and IL-18 release and can drive pyroptotic cell death (23–25). Furthermore, mitochondrial dysfunction and impaired mitophagy contribute to elevated DAMPs (e.g., reactive oxygen species, ROS) and the accumulation of dysfunctional mitochondria or mitochondrial DNA (mtDNA) leakage, all potent activators of NLRP3 inflammasomes (26). Thus, inflammasome activation and its intersection with inflammatory cell death pathways, including PANoptosis, can profoundly shape host responses to infection and tissue damage.

In the CNS, microglia, astrocytes, and neurons all express NLRP3, and its dysregulated activation contributes to the pathogenesis of neurodegenerative diseases, autoimmune disorders, and viral encephalitis (27, 28). Several neurotropic viruses, including influenza A virus and SARS-CoV-2, are known to engage the NLRP3 inflammasome through diverse mechanisms, underscoring its central role in viral neuropathogenesis (29–31). Thus, dysregulated NLRP3 signaling represents a critical nexus in virus-induced neuroinflammation. In this study, we investigated how PHEV engages inflammasome signaling and provide evidence that the viral N protein promotes NLRP3 inflammasome assembly and contributes to neuroinflammatory injury and PANoptosis-associated cell-death signaling. These findings identify a virus-host interface that may be amenable to therapeutic targeting in neurotropic coronavirus-associated disease.

## RESULTS

### PHEV induces widespread neuroinflammatory responses in mice

**Fig 1:**
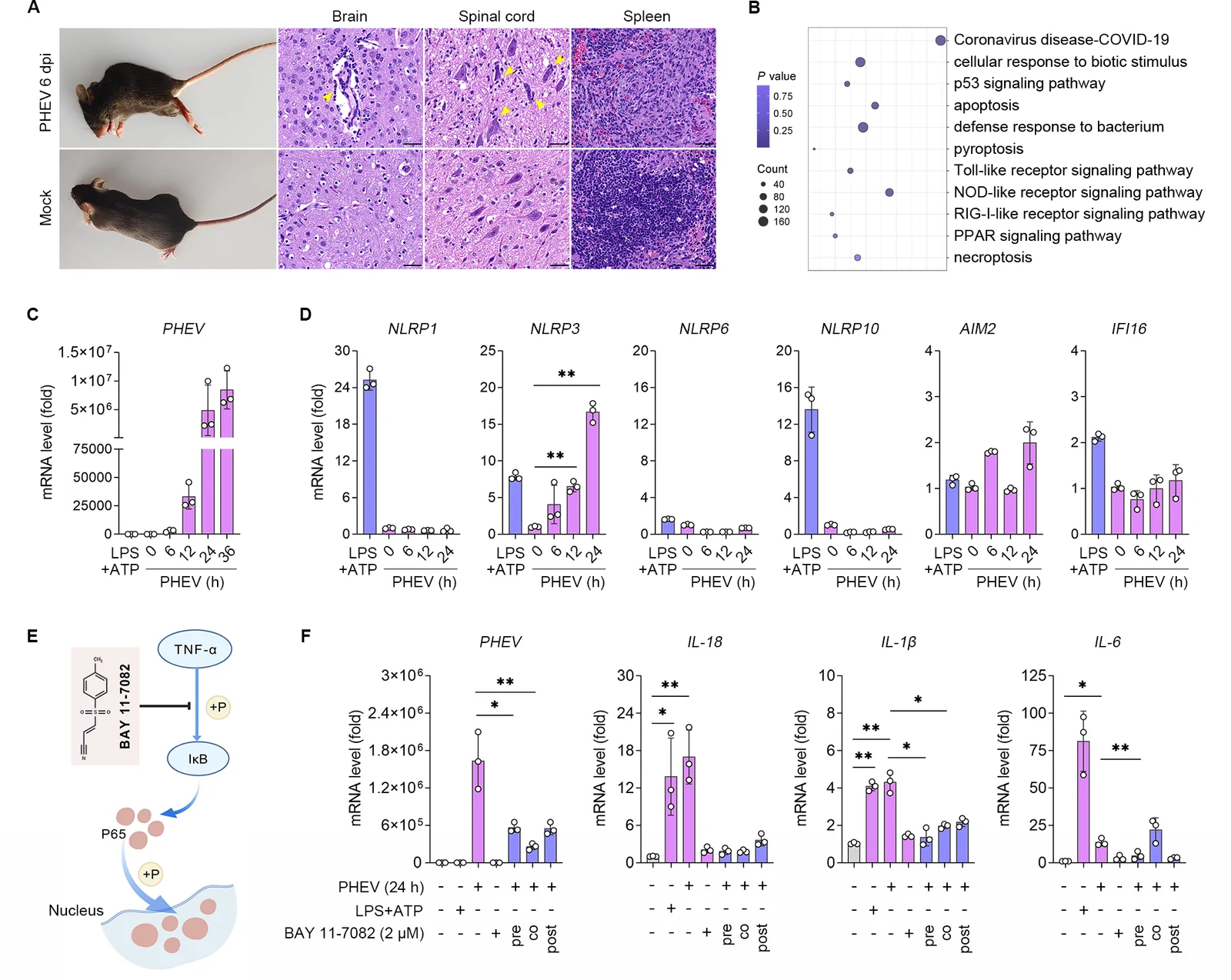
PHEV infection triggers robust systemic inflammation. (**A**) Clinical manifestations and histopathology in PHEV-infected mice at 6 days post-infection (dpi). Four-week-old C57BL/6 mice were intranasally inoculated with PHEV. Representative images show H&E-stained sections of the brain (arrowheads indicate perivascular cuffing) and spinal cord (arrowheads indicate neuronophagia). Scale bars, 50 µm. (**B**) Enrichment analysis of differentially expressed genes (DEGs) in brain tissue at 6 dpi. The heatmap displays hierarchical clustering of significantly enriched GO terms and KEGG pathways (*n* = 66 DEGs). Color intensity reflects −log₁₀(*P*-value) from Fisher’s exact test. (**C and D**) PHEV and indicated gene mRNA expression in PHEV-infected RAW264.7 macrophages. Positive controls received LPS priming (1 μg/mL; 6 h), followed by ATP stimulation (2.5 mM; 30 min). (**E**) Effect of the NF-κB inhibitor BAY 11-7082 on NF-κB signaling. (**F**) RAW264.7 cells were treated with 2 µM BAY 11-7082 either pre-treatment (pre; 1 h before infection), co-treatment (co; simultaneously with infection), or post-treatment (post; 1 h after infection). The supernatants were collected for the measurement of PHEV, IL-1β, IL-18, and IL-6 mRNA levels. Normalized to GAPDH using the 2^–ΔΔCt^ method. LPS + ATP served as a positive control. Data are represented as mean ± SD, obtained from three independent biological replicates; *P  <  0.05, **P  <  0.01 (unpaired two-tailed *t*-test).

It has been established that PHEV causes acute encephalitis in young pigs in both clinical cases and experimental infections. To model this pathology, 4-week-old C57BL/6 mice were intranasally inoculated with PHEV. Within 6 days post-infection, the animals developed severe CNS impairment, characterized by lethargy, weight loss, hunched posture, ruffled fur, limb paralysis, and paddling movements, which ultimately led to mortality within 6 days post-infection (Fig. 1A). Histopathological examination of infected mice revealed pronounced inflammatory cell infiltration in the brain, manifested as perivascular cuffing, a hallmark of neuroinflammation. Extensive neuronal necrosis was observed in the spinal cord (Fig. 1A). Consistent with our previous report (15), the 6 dpi time point was selected for RNA sequencing analysis to capture the transcriptional signature associated with fully established neuroinflammation and maximal viral burden, which correlates with severe clinical outcome and histopathological damage. Enrichment analysis of differentially expressed genes highlighted significant involvement of pathways related to innate immune signaling and inflammatory cell death, including NOD-like receptor signaling, necroptosis, RIG-I-like receptor signaling, and Toll-like receptor signaling (Fig. 1B). These findings implicated inflammasome-associated signaling in PHEV-induced CNS disease.

To identify a suitable myeloid model for mechanistic studies, we compared PHEV infection in primary alveolar macrophages (PAMs), RAW264.7 macrophages, and BV2 microglia. RAW264.7 cells were markedly more permissive, exhibiting ~350-fold higher viral burden than PAMs at 24 hpi (Fig. 1C; Fig. S1A through E), and were therefore used for subsequent analysis of inflammatory signaling. In RAW264.7 cells, PHEV robustly induced NLRP3 expression (Fig. 1D) and activated NF-κB signaling (Fig. S1F). To functionally interrogate the contribution of NF-κB-driven inflammation, we employed BAY 11-7082 (Fig. 1E), an inhibitor of IκB-α phosphorylation that attenuates NF-κB activation and subsequent pro-inflammatory cytokine production (32). Concentration-dependent cytotoxicity assessment using the CCK-8 assay identified 2 μM as a non-toxic working concentration for subsequent experiments (Fig. S1G). Pre-treatment, co-treatment, or post-treatment of RAW264.7 cells with BAY 11-7082 significantly reduced PHEV infection and suppressed the induction of key pro-inflammatory cytokines, including IL-1β, IL-18, and IL-6 (Fig. 1F). Collectively, these findings demonstrate that PHEV infection triggers a potent neuroinflammation response and that its pharmacological attenuation reduces viral infectivity and cytokine storm, underscoring the critical role of inflammation in PHEV pathogenesis.

### NLRP3 inflammasome is essential for PHEV-induced inflammatory and viral propagation

**Fig 2:**
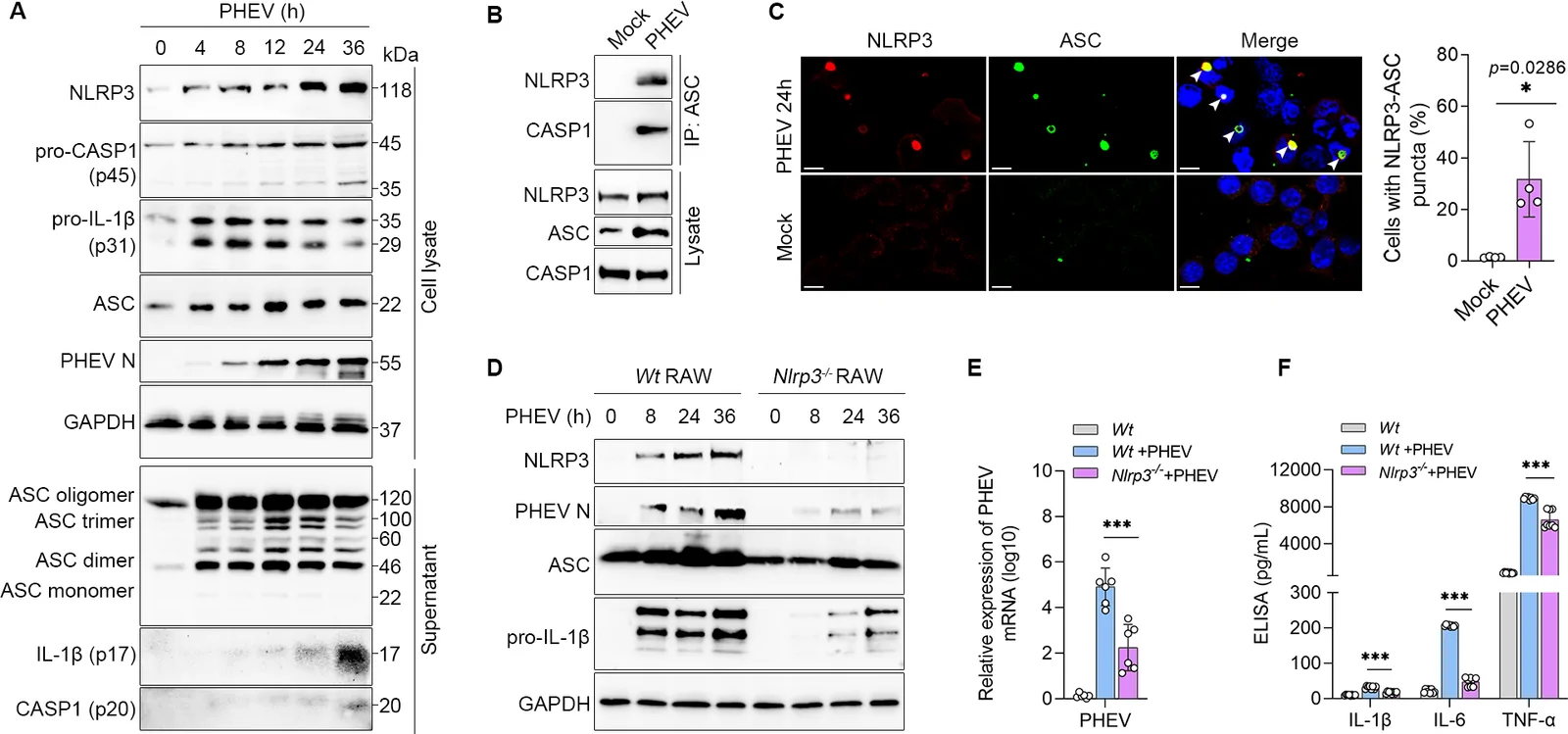
PHEV activates the NLRP3 inflammasome in RAW264.7 macrophages. (**A**) Immunoblot analysis of NLRP3 inflammasome components in RAW264.7 cells infected with PHEV (MOI = 1) for the indicated times. Lysates were probed for NLRP3, pro-caspase-1 (pro-CASP1), cleaved CASP1 (P20), pro-IL-1β, and mature IL-1β (P17). ASC oligomerization (cross-linked pellet) indicates inflammasome assembly. (**B**) Co-immunoprecipitation of ASC-associated complexes from PHEV-infected (24 h) RAW264.7 cell lysates. Cell lysates were immunoprecipitated using an anti-ASC antibody. (**C**) Confocal microscopy of RAW264.7 cells infected with PHEV for 24 h, stained for ASC (green) and NLRP3 (red). Nuclei were counterstained with Hoechst (blue). Scale bars, 10 μm. (**D and E**) Viral replication in wild-type (Wt) and *Nlrp3^−/−^* RAW264.7 macrophages. PHEV N protein levels were assessed by immunoblotting (**D**), and viral mRNA was quantified by qRT-PCR (**E**) at 24 h post-infection. Relative mRNA expression was normalized to GAPDH using the 2^–ΔΔCt^ method. (**F**) Secretion of IL-1β, IL-6, and TNF-α from the supernatants of PHEV-infected Wt and *Nlrp3^−/−^* RAW264.7 macrophages measured by ELISA at 24 h. Data are mean ± SD of eight independent experiments. *P < 0.05, **P < 0.01, ***P < 0.001 (unpaired two-tailed *t*-test).

Given the central role of the NLRP3 inflammasome in orchestrating inflammatory responses to viral infections, including those caused by coronaviruses (10, 33), we sought to determine whether PHEV directly engages this pathway. In PHEV-infected RAW264.7 macrophages, we observed a progressive accumulation of NLRP3 and its inactive zymogen, pro-caspase-1 (pro-CASP1 or p45). This was accompanied by the proteolytic cleavage of pro-CASP1 into its active p20 fragment (p20) and the maturation of IL-1β to its p17 form (p17), indicative of canonical inflammasome activation (Fig. 2A). A definitive hallmark of NLRP3 activation is the prion-like polymerization of the adaptor protein ASC into micron-sized aggregates termed ASC specks, which nucleate CASP1 autoactivation (34). PHEV infection robustly induced ASC oligomerization (Fig. 2A). A co-immunoprecipitation assay further demonstrated enhanced interactions among the core inflammasome components NLRP3, ASC, and CASP1 in infected cells (Fig. 2B). Consistently, confocal microscopy revealed a significant increase in the formation of distinct cytoplasmic ASC specks following PHEV challenge (Fig. 2C).

To determine the functional contribution of NLRP3, we utilized isogenic NLRP3-knockout (*Nlrp3^−/−^*) RAW264.7 macrophages. Genetic ablation of NLRP3 markedly impaired IL-1β maturation and, notably, substantially reduced PHEV replication, evidenced by reduced viral mRNA and N protein levels (Fig. 2D and E). Reduced viral propagation was also observed in N2a cells (Fig. S2A), but to a lesser extent than that in macrophages, due to cell-specific inflammasome activity. Moreover, profiling demonstrated that the inflammatory output downstream of NLRP3 was significantly attenuated, with lower secretion of IL-1β, IL-6, and TNF-α from PHEV-infected *Nlrp3^−/−^* macrophages compared to wild-type (Wt) controls (Fig. 2F). Taken together, these findings establish that PHEV infection actively assembles and activates the NLRP3 inflammasome, which functions not only as a passive sensor but also as a crucial host factor that facilitates both viral replication and the ensuing inflammatory cascade.

### PHEV nucleocapsid protein promotes NLRP3 inflammasome assembly in reconstituted systems

**Fig 3:**
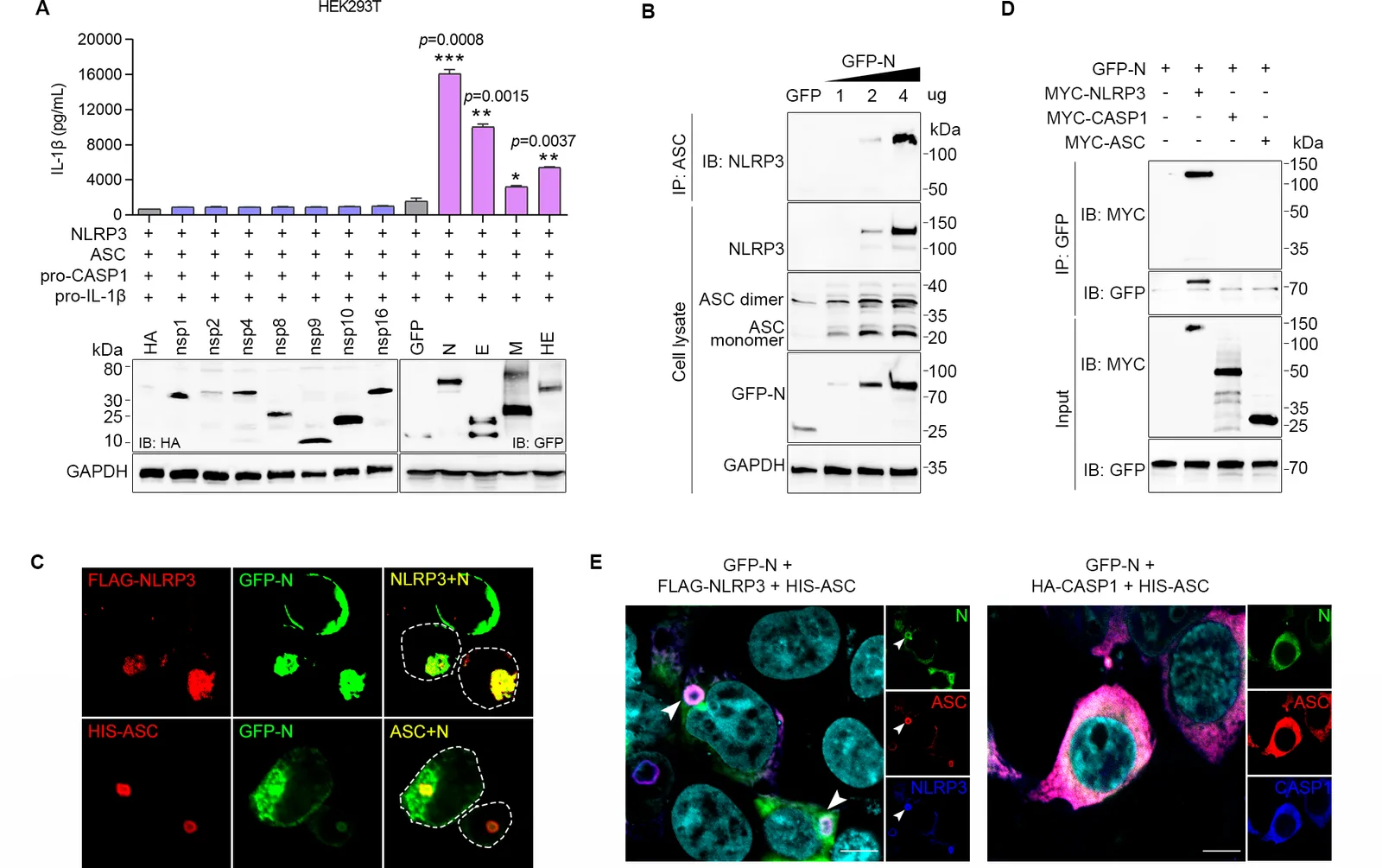
PHEV N protein is involved in the NLRP3 inflammasome assembly. (**A**) Reconstitution of the NLRP3 inflammasome in HEK293T cells. The cells were co-transfected with NLRP3, ASC, pro-CASP1, and pro-IL-1β, together with plasmids expressing the indicated PHEV non-structural (HA-tagged) or structural (GFP-tagged) proteins. The secreted mature IL-1β was quantified by ELISA (top). The expression of the transfected proteins was verified by immunoblotting (bottom). (**B**) Dose-dependent enhancement of NLRP3-ASC interaction by the PHEV N protein. HEK293T cells were transfected with increasing amounts of GFP-N plasmid, and then, cell lysates were subjected to immunoprecipitation with an anti-ASC antibody. ASC oligomerization was assessed in cross-linked pellets. (**C**) Subcellular co-localization of the PHEV N protein with NLRP3 and ASC. HEK293T cells expressing GFP-N, together with MYC-NLRP3 (top) or HIS-ASC (bottom), were analyzed by confocal microscopy. Nuclei were stained with Hoechst (blue). (**D**) Interaction between the PHEV N protein and the NLRP3 inflammasome. HEK293T cells were co-transfected with GFP-N and MYC-tagged NLRP3, ASC, or CASP1. Lysates were immunoprecipitated with anti-MYC or anti-GFP antibodies and analyzed as indicated. (**E**) HEK293T cells were transfected with GFP-N, HIS-ASC, and either FLAG-NLRP3 (left) or HA-CASP1 (right). Representative confocal images show co-localization in cytoplasmic aggregates (specks). Nuclei are shown in cyan. Scale bars, 10 µm. Data are represented as the mean ± SD, obtained from three independent biological replicates; *P  <  0.05, **P  <  0.01, ***P  <  0.001 vs control (unpaired two-tailed *t*-test).

To identify the viral factor responsible for inflammasome engagement, we reconstituted an NLRP3 inflammasome in HEK293T cells by co-expressing the core components NLRP3, ASC, and pro-CASP1 together with the substrate pro-IL-1β. This reconstituted sensing platform was then used to screen individual PHEV proteins, including non-structural proteins nsp1, nsp2, nsp4, nsp8, nsp9, nsp10, and nsp16, as well as the structural proteins N, E, M, and HE. Measurement of secreted IL-1β showed that several PHEV proteins exhibited measurable activity, with the N protein inducing the strongest release of mature IL-1β (Fig. 3A). Notably, the E protein also increased IL-1β secretion, although to a lesser extent than N, suggesting that multiple viral proteins may contribute to inflammasome activation through distinct mechanisms.

We therefore prioritized N for mechanistic analysis because it displayed the strongest activity in the screening assay and enhanced the endogenous association between NLRP3 and oligomerized ASC in a dose-dependent manner (Fig. 3B). Confocal microscopy further showed that the PHEV N protein extensively co-localized with both NLRP3 and its adaptor ASC, and all were recruited into large cytoplasmic aggregates (specks) characteristic of inflammasome activation (Fig. 3C). Consistent with these findings, co-immunoprecipitation assays demonstrated that PHEV N interacted with NLRP3 in co-transfected HEK293T cells, whereas no detectable interaction with ASC or CASP1 was observed (Fig. 3D). Moreover, co-expression of NLRP3, ASC, and N promoted the coalescence of all three components into a distinct circular superstructure, a pattern not observed when CASP1 was co-expressed with ASC and N (Fig. 3E).

By comparison, although E enhanced inflammasome output and promoted ASC complex formation (Fig. S3B and C), it did not show detectable binding to NLRP3 in co-immunoprecipitation assays (Fig. S3D). These data suggest that PHEV E may facilitate inflammasome activation through a mechanism distinct from that of N, potentially analogous to the viroporin-dependent disruption of intracellular Ca²^+^ homeostasis previously reported for SARS-CoV E protein (35). Together, these findings identify the PHEV N protein as a viral factor that directly engages NLRP3 and promotes assembly of the NLRP3–ASC signaling scaffold in reconstituted cells.

### The C-terminal domain of PHEV N protein binds to the NLRP3 PYD domain

**Fig 4:**
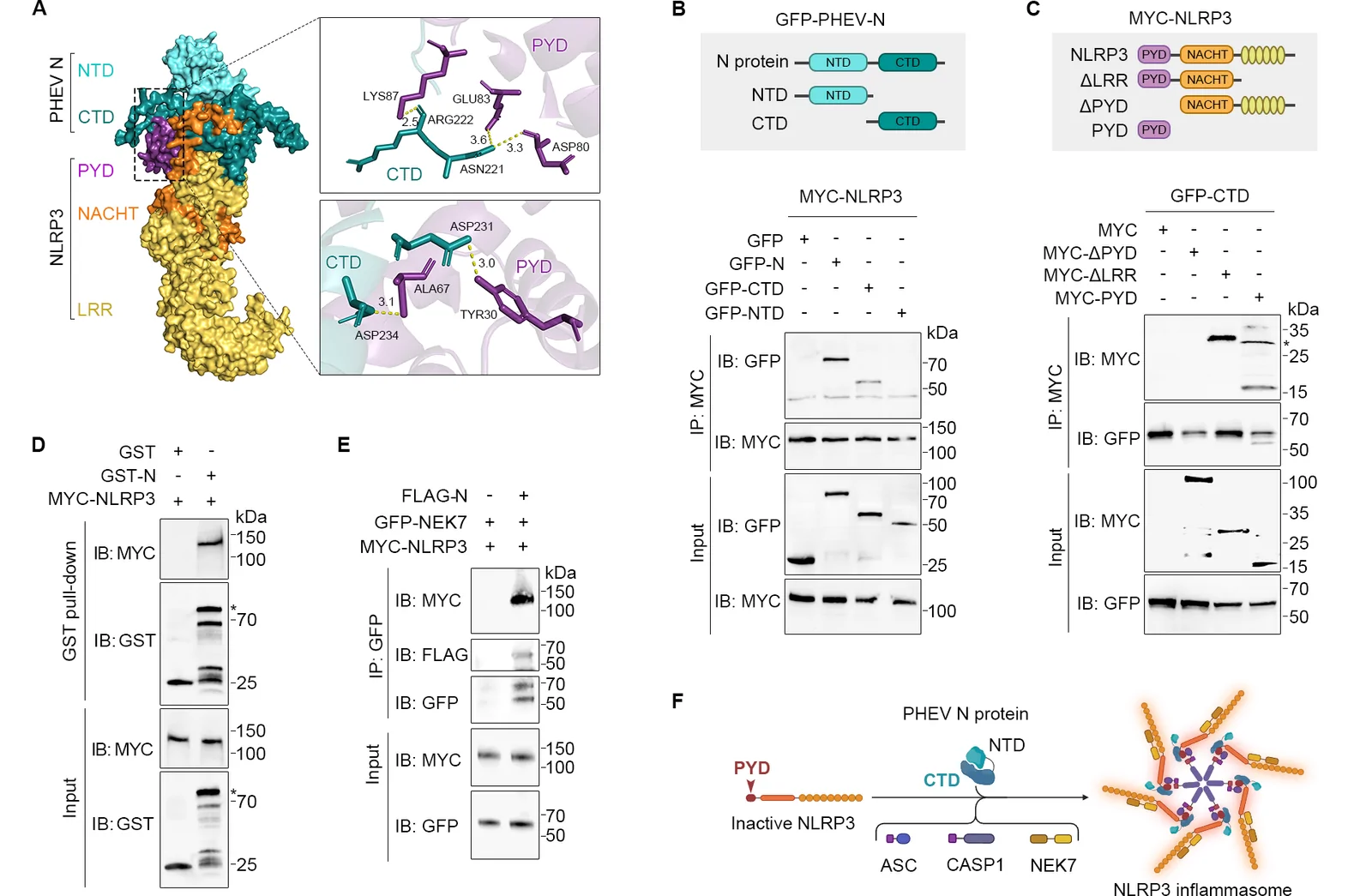
The CTD domain of the PHEV N protein interacts with the PYD domain of NLRP3. (**A**) Predicted structural interaction between the PHEV N protein (homology-modeled, SWISS-MODEL) and NLRP3 (AlphaFold prediction). Molecular docking (HDOCK) reveals potential binding interfaces; zoom-in shows key interacting residues (colored using PyMOL). (**B**) Co-IP in HEK293T cells co-expressing MYC-NLRP3 with full-length GFP-N, its N-terminal domain (NTD), or C-terminal domain (CTD). Cell lysates were immunoprecipitated using anti-MYC antibody and analyzed using anti-GFP and anti-MYC antibodies. (**C**) Co-IP in HEK293T cells co-expressing GFP-CTD with MYC-tagged NLRP3 mutants, including ΔPYD, ΔLRR, and PYD domain. Cell lysates were immunoprecipitated using the anti-GFP antibody and analyzed using the anti-GFP and anti-MYC antibodies. (**D**) GST pull-down assays. Purified GST or GST-tagged PHEV N protein was incubated with lysates from HEK293T cells expressing MYC-NLRP3. (**E**) Co-immunoprecipitation analysis indicated that PHEV N expression does not interfere with the NLRP3-NEK7 interaction in HEK293T cells. (**F**) Schematic model depicting the CTD-PYD interaction as a direct trigger for NLRP3 inflammasome assembly and activation.

Having established that the PHEV N protein interacts with NLRP3 to promote inflammasome assembly, we next sought to map the precise interacting domains. The PHEV N protein comprises two principal domains: an N-terminal domain (NTD) and a C-terminal domain (CTD), responsible for RNA binding and oligomerization, respectively (36). NLRP3 contains three functional domains: an N-terminal pyrin domain (PYD), a central NACHT domain, and a C-terminal leucine-rich repeat (LRR) domain (37). A homology model of the PHEV N protein was docked against an AlphaFold-derived structure of NLRP3 using GRAMM software. Molecular docking predicted a stable interaction interface, revealing potential salt bridges and hydrogen bonds formed between the CTD of the N protein and the PYD (residues 1–93) of NLRP3 (Fig. 4A). Co-immunoprecipitation assays in HEK293T cells confirmed that full-length NLRP3 specifically bound the isolated CTD, but not the NTD, of the PHEV N protein, validating the computational model (Fig. 4B). To identify the NLRP3 domain responsible for this interaction, we constructed a panel of domain-specific variants: a PYD-deletion mutant (ΔPYD), an LRR-deletion mutant (ΔLRR), and an isolated PYD domain (Fig. 4C). HEK293T cells co-expressing the PHEV N-CTD with each NLRP3 variant further demonstrated a specific interaction between the viral N-CTD and the NLRP3 PYD domain (Fig. 4C). GST pull-down assays further confirmed a direct interaction between the PHEV N protein and NLRP3 (Fig. 4D), consistent with the finding that the N-CTD specifically targets the PYD domain of NLRP3.

A critical checkpoint in NLRP3 inflammasome activation is the recruitment of the kinase NEK7, which binds the LRR domain to license full complex assembly (21). We therefore investigated whether the PHEV N protein interferes with this essential host factor interaction. Co-immunoprecipitation experiments revealed that the presence of the N protein did not impede the NLRP3-NEK7 interaction (Fig. 4E). These findings indicate the CTD of the viral N protein could engage the NLRP3 PYD and delineate a specific interaction central to PHEV-induced inflammasome activation. This binding initiates the assembly of the NLRP3-ASC platform without interfering with the NEK7-dependent activation step (Fig. 4F). To explore whether this domain-specific interaction might extend beyond PHEV, we performed additional co-immunoprecipitation assays using SARS-CoV-2 N domain constructs. Similar to PHEV N, the CTD of SARS-CoV-2 N specifically interacted with the PYD domain of NLRP3 (Fig. S2E). These data suggest that the N protein acts as a pro-inflammatory viral scaffold and that CTD-mediated engagement of NLRP3 PYD may represent a shared mechanism among at least some coronaviral nucleocapsid proteins.

### Pharmacological inhibition of NLRP3 restricts PHEV replication and pathogenesis

**Fig 5:**
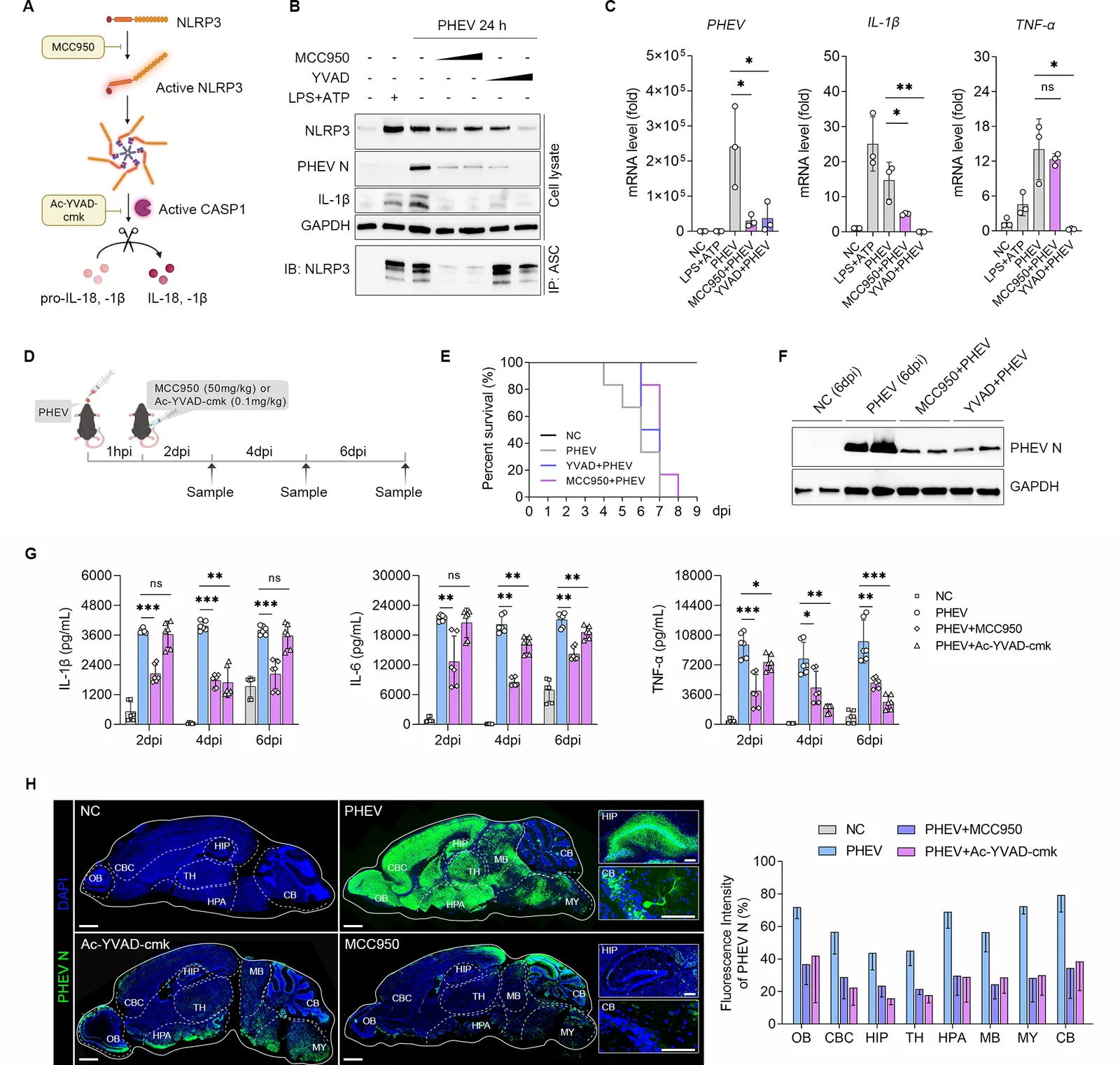
Pharmacological inhibition of NLRP3 inflammasome restricts PHEV infection. (**A**) Schematic of NLRP3 inhibitor MCC950 (targeting NLRP3 ATPase) and CASP1 inhibitor Ac-YVAD-cmk. (**B and C**) RAW264.7 cells were infected with PHEV and treated with MCC950 (5 or 10 nM), Ac-YVAD-cmk (40 or 80 μM), or LPS + ATP (positive control). Viral N protein levels were assessed by immunoblotting (**B**), and the indicated mRNA was quantified by qRT-PCR (**C**). Relative mRNA expression was normalized to GAPDH using the 2^–ΔΔCt^ method. (**D**) Experimental timeline for *in vivo* treatment conditions. C57BL/6 mice (*n* = 6/group) were intranasally infected with PHEV, followed by intraperitoneal injection of MCC950 (50 mg/kg) or Ac-YVAD-cmk (0.1 mg/kg). Mice were sacrificed at 2, 4, and 6 dpi. (**E**) Survival curves of PHEV-infected mice treated with vehicle, MCC950, or Ac-YVAD-cmk over 9 days. (**F**) Immunoblot analysis of the PHEV N protein in brain homogenates at 6 dpi. (**G**) Levels of IL-1β, IL-6, and TNF-α in brain supernatants were measured by ELISA at 6 dpi. (**H**) Representative immunofluorescence images of brain sections stained for PHEV antigen (green) and nuclei (Hoechst, blue) at 6 dpi. Scale bars: 200 µm in the zoomed hippocampal (HIP) regions; 100 µm in the zoomed cerebellar (CB) regions. Data are mean ± SD (*n* = 3 for *in vitro*, *n* = 6 for *in vivo*); *P < 0.05, **P < 0.01, ***P < 0.001 vs vehicle group (unpaired two-tailed *t*-test).

To define the functional contribution of the NLRP3 inflammasome to PHEV infection, we employed specific pharmacological inhibitors targeting key nodes of this pathway (Fig. 5A). MCC950, a selective small-molecule inhibitor, binds directly to the NLRP3 NACHT domain to block its ATPase activity and prevent inflammasome assembly (37). In a complementary approach, we employed Ac-YVAD-cmk, a cell-permeable inhibitor that selectively targets CASP1, thereby inhibiting the processing of IL-1β or IL-18 maturation and pyroptosis. Pre-treatment of PHEV-infected RAW264.7 cells with either MCC950 or Ac-YVAD-cmk significantly reduced viral replication, as evidenced by decreased viral mRNA and N protein levels (Fig. 5B and C; Fig. S3A and B). This was accompanied by a marked suppression of pro-inflammatory cytokine release (Fig. 5C). The protective effect against viral propagation and inflammation paralleled that observed with the NF-κB inhibitor BAY 11-7082 (Fig. 1F), highlighting the integrated role of these signaling hubs in PHEV pathogenesis.

We next assessed the therapeutic potential of this strategy *in vivo*. C57BL/6 mice were infected intranasally with PHEV and treated intraperitoneally with MCC950 or Ac-YVAD-cmk according to established regimens (Fig. 5D) (38, 39). Both inhibitors delay disease progression compared to vehicle-treated, infected controls (Fig. 5E). Analysis of brain tissues at the typical peak of disease (6 dpi) revealed that inhibitor treatment substantially reduced viral protein and loads (Fig. 5F; Fig. S3C) and attenuated the production of inflammatory cytokines, including IL-1β, IL-6, and TNF-α (Fig. 5G). Corroborating these results, fluorescence microscopy of brain sections showed a clear decrease in viral antigen presence in the CNS following inhibitor administration (Fig. 5H). These data demonstrate that disruption of NLRP3 inflammasome activity not only mitigates neuroinflammation but also restricts PHEV replication, highlighting the inflammasome as a critical host factor co-opted by the coronavirus to enhance its propagation.

### NLRP3 deficiency confers resistance to PHEV-induced neuroinflammation and mortality

**Fig 6:**
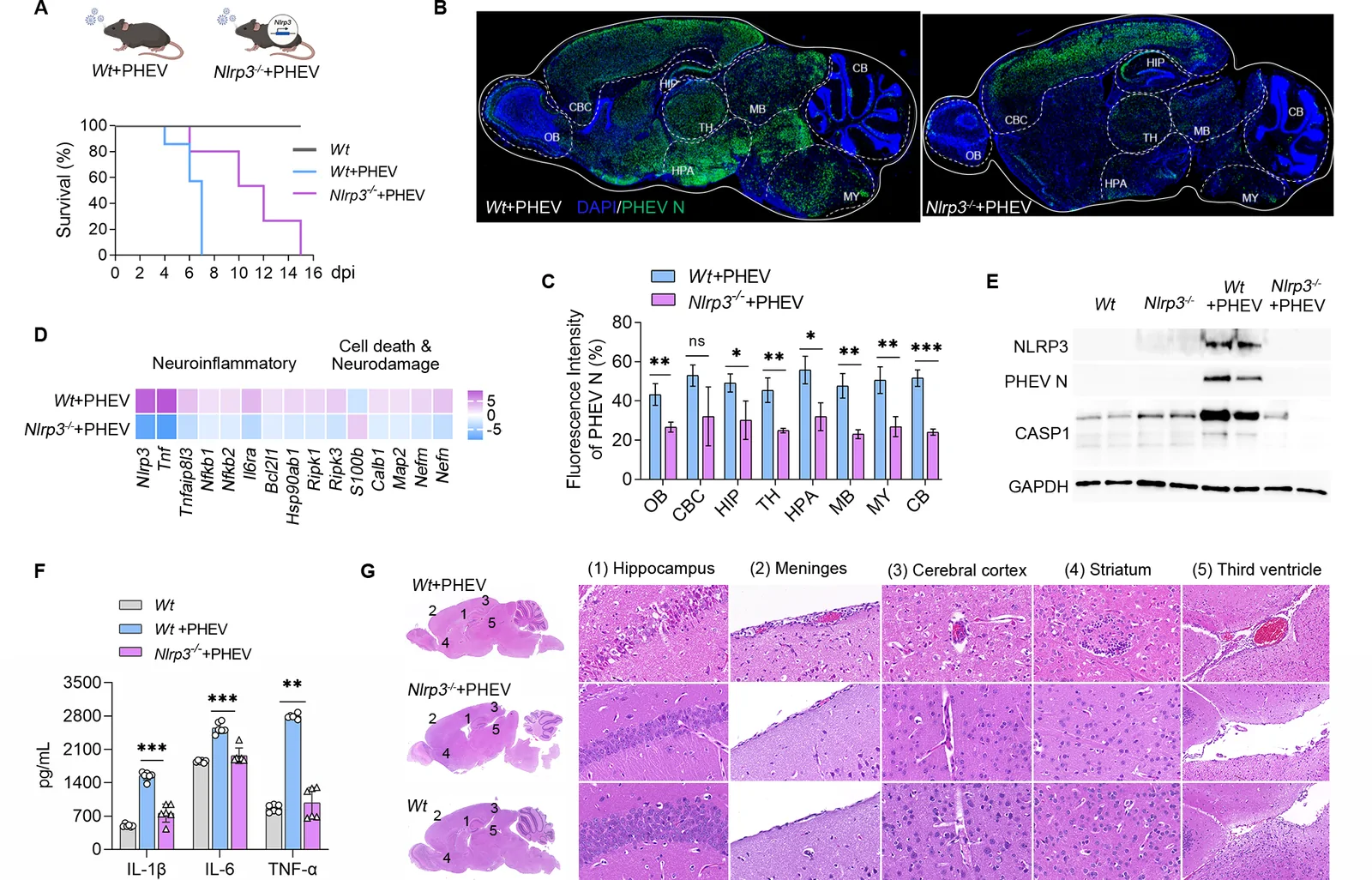
NLRP3 deficiency protects mice from PHEV-induced neuroinflammation and mortality. (**A**) Experimental model and survival curves of PHEV-infected Wt and *Nlrp3^−/−^* mice over 16 days (*N* = 6/group). (**B**) Representative brain sections from infected mice at 6 dpi, stained for PHEV N (green) and nuclei (Hoechst, blue). (**C**) Quantification of N fluorescence intensity in (**B**), analyzed with ImageJ. (**E**) Immunoblot analysis of PHEV N protein levels in brain homogenates from Wt and *Nlrp3^−/−^* mice at 6 dpi. (**D**) Heat map of RNA-Seq data from brain tissues at 6 dpi, highlighting DEGs related to neuroinflammation and neural injury in Wt vs *Nlrp3^−/−^* mice. (**F**) Concentrations of IL-1β, IL-6, and TNF-α in brain supernatants at 6 dpi, measured by ELISA. (**G**) Representative H&E-stained brain sections (hippocampus, meninges, cortex, striatum, and ventricle) from Wt and *Nlrp3^−/−^* mice at 6 dpi, showing attenuated neuropathology in knockout mice. Scale bars: 50 µm in the zoomed hippocampal, meningeal, cerebral cortical, and striatal regions; 200 µm in the zoomed third ventricular regions. Data are represented as the mean ± SD, obtained from five independent biological replicates; *P  <  0.05, **P  <  0.01, ***P  <  0.001 (unpaired two-tailed *t*-test).

The pivotal role of NLRP3 in PHEV pathogenesis was further substantiated using C57BL/6 mice harboring a germline deletion of the *Nlrp3* gene (*Nlrp3^−/−^* mice). Upon intranasal PHEV infection, *Nlrp3^−/−^* mice showed prolonged survival and delayed mortality compared with their wild-type counterparts, together with a marked reduction in the CNS viral load (Fig. 6A; Fig. S3D). Immunofluorescence staining analysis showed an overall reduction in PHEV antigen distribution across multiple brain regions in *Nlrp3^−/−^* mice, including the olfactory bulb (OB), hippocampus (HIP), thalamus (TH), hypothalamus (HPA), midbrain (MB), medulla oblongata (MY), and cerebellum (CB) (Fig. 6B and C). Notably, the reduced viral burden in *Nlrp3^−/−^* mice was accompanied by attenuated neuroinflammation. For example, in the thalamus of PHEV-infected *Nlrp3^−/−^* mice, the mean fluorescence intensity of viral antigen was decreased by 32.99% ± 0.3218%, in parallel with weaker microglial and astrocytic activation (Fig. S3E and F). In contrast, in the cerebral cortex (CBC), viral antigen levels did not differ significantly between Wt and *Nlrp3^−/−^* mice, and microglial activation was likewise not significantly altered (Fig. S3E and F). These findings support an association between regional viral propagation and neuroinflammatory responses in the CNS.

Transcriptomic profiling of brain tissues at the peak of disease (6 dpi) revealed profoundly divergent host transcriptional responses between Wt and *Nlrp3^−/−^* mice. Differential expression analysis identified a cohort of neuroinflammation-related genes (i.e., *Nlrp3, Tnf*, *Tnfaip8l3*, *Nfkb1*, *Nfkb2*, and *Il6ra*) and neural injury-associated genes (i.e., *Ripk1, Ripk3*, *S100b*, *Calb1*, *Map2*, and *Nefm*) that were robustly upregulated in Wt mice but blunted in *Nlrp3^−/−^* mice (Fig. 6D). Consistent with these transcriptomic findings, immunoblot analysis further showed reduced expression of NLRP3 and its downstream effector CASP1 in the brain homogenates of *Nlrp3^−/−^* mice following PHEV infection (Fig. 6E). This attenuated inflammatory signature aligned with a significant decrease in the production of key cytokines, including IL-1β and TNF-α, in the brains of infected knockout animals (Fig. 6F). Histopathological evaluation further linked NLRP3 activation to CNS damage. Brain sections from PHEV-infected Wt mice exhibited severe neuropathology, including neuronal necrosis in the hippocampal granule cell layer, meningeal inflammatory infiltrates indicative of meningitis, cortical perivascular cuffing and edema, striatal glial nodules, and third ventricular congestion (Fig. 6G). Conversely, PHEV-infected *Nlrp3^−/−^* mice presented with markedly preserved neuroarchitecture, showing minimal pyramidal cell loss, scarce perivascular cuffing, and an absence of overt inflammatory lesions (Fig. 6G). Attenuated inflammation in the knockout background was further confirmed by reduced IL-1β immunoreactivity (Fig. S3G). Collectively, these *in vivo* findings indicate that NLRP3 deficiency reduces the CNS viral burden and mitigates the inflammatory cascade associated with neural injury, supporting a role for NLRP3 in accelerating PHEV-induced neuroinflammation and disease progression.

### NLRP3 inflammasome contributes to PHEV-induced mitochondrial dysfunction and PANoptosis

**Fig 7:**
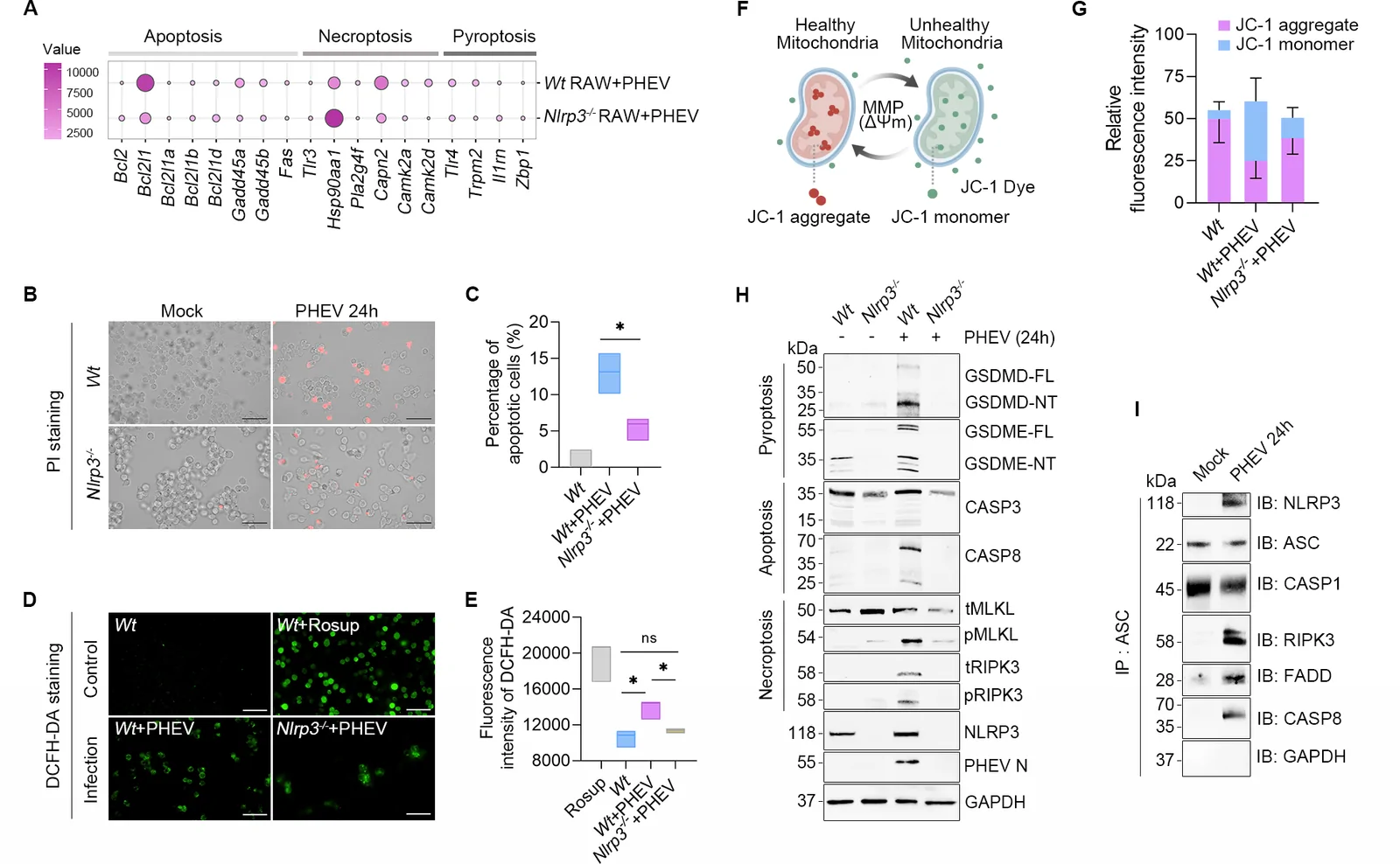
The NLRP3 inflammasome drives PHEV-induced mitochondrial dysfunction and PANoptosis. (**A**) Bubble plot of DEGs in Wt vs *Nlrp3^−/−^* RAW264.7 macrophages 24 h after PHEV infection, highlighting 18 core genes regulating PANoptosis (FDR  ≤  0.001, DEGseq). (**B and C**) Cell death measured by PI staining (**B**) and quantified by flow cytometry (**C**) in Wt and *Nlrp3^−/−^*  macrophages infected with PHEV for 24 h. Scale bar, 50 µm. (**D and E**) ROS production detected by DCFH-DA staining (**D**) and quantified (**E**) in infected macrophages. ROSUP served as a positive control. Scale bar, 50 µm. (**F**) Schematic of the JC-1 assay for ΔΨm. (**G**) Quantification of ΔΨm loss (JC-1 red/green ratio) in Wt and *Nlrp3^−/−^* macrophages after PHEV infection. (**H**) Immunoblot analysis of PANoptosis-related proteins in Wt and *Nlrp3^−/−^* macrophages infected with PHEV for 0 or 24 h. (**I**) RAW264.7 cells were treated with PHEV for 24 h, and the cell lysates were immunoprecipitated using anti-ASC antibody and analyzed using anti-RIPK1, anti-RIPK3, anti-FADD, anti-CASP1, anti-CASP8, anti-NLRP3, and anti-ASC antibody. Data are represented as the means ± SD, *n* = 3; *P < 0.05 (unpaired two-tailed *t*-test).

PANoptosis represents a coordinated cell death pathway that integrates the features of pyroptosis, apoptosis, and necroptosis, with the NLRP3 inflammasome serving as a key upstream activator capable of initiating this process‌‌ (23). Transcriptomic analysis of PHEV-infected Wt versus *Nlrp3^−/−^* RAW264.7 macrophages revealed differential expression of 18 genes coregulating PANoptosis (Fig. 7A). Corroborating this, quantification of cell death by propidium iodide (PI) staining demonstrated a significant increase in PHEV-infected Wt cells, which was markedly attenuated in *Nlrp3^−/−^* cells (Fig. 7B). Flow cytometric analysis with annexin V/PI further confirmed that the rise in apoptotic cell populations following infection was substantially reduced in the absence of NLRP3 (Fig. 7C). Given the central role of mitochondrial integrity in cell death decisions (40), we assessed mitochondrial function by detection of intracellular reactive oxygen species (ROS) using a 2′,7′-dichlorodihydrofluorescein diacetate (DCFH-DA) assay. PHEV infection led to a progressive accumulation of intracellular ROS in Wt cells and peaked at 24 hpi (Fig. S3H), whereas this ROS surge was significantly blunted in *Nlrp3^−/−^* cells (Fig. 7D and E). Consistently, pretreatment with the mitochondrial ROS scavenger MitoTEMPO partially reduced ROS accumulation in PHEV-infected cells (Fig. S3I), supporting the presence of mitochondrial oxidative stress in this model. Mitochondrial damage was further evidenced by a loss of ΔΨm, detected by a shift in JC-1 fluorescence from red aggregates to green monomers (Fig. 7F). This dissipation of ΔΨm was pronounced in infected Wt cells but markedly attenuated in *Nlrp3^−/−^* cells (Fig. 7G). These findings indicate that PHEV infection is associated with mitochondrial oxidative stress and dysfunction and that NLRP3 contributes to the magnitude of these mitochondrial alterations.

To dissect the specific death modalities involved, we next examined the execution of pyroptosis, apoptosis, and necroptosis. Immunoblot analysis showed that PHEV-induced cleavage of gasdermin D (GSDMD) and gasdermin E (GSDME) was diminished in *Nlrp3^−/−^* cells, indicating that pyroptosis is NLRP3-dependent (Fig. 7H). Interestingly, NLRP3 deficiency shifted the cell death profile, with signatures more indicative of necroptosis becoming apparent (Fig. 7H). Finally, co-immunoprecipitation confirmed that PHEV infection promotes the assembly of a macromolecular complex containing NLRP3, ASC, Fas-associated via death domain (FADD), receptor-interacting serine/threonine kinase 1 (RIPK1), and RIPK3, which are core components characteristic of a functional PANoptosome (Fig. 7I).

**Fig 8:**
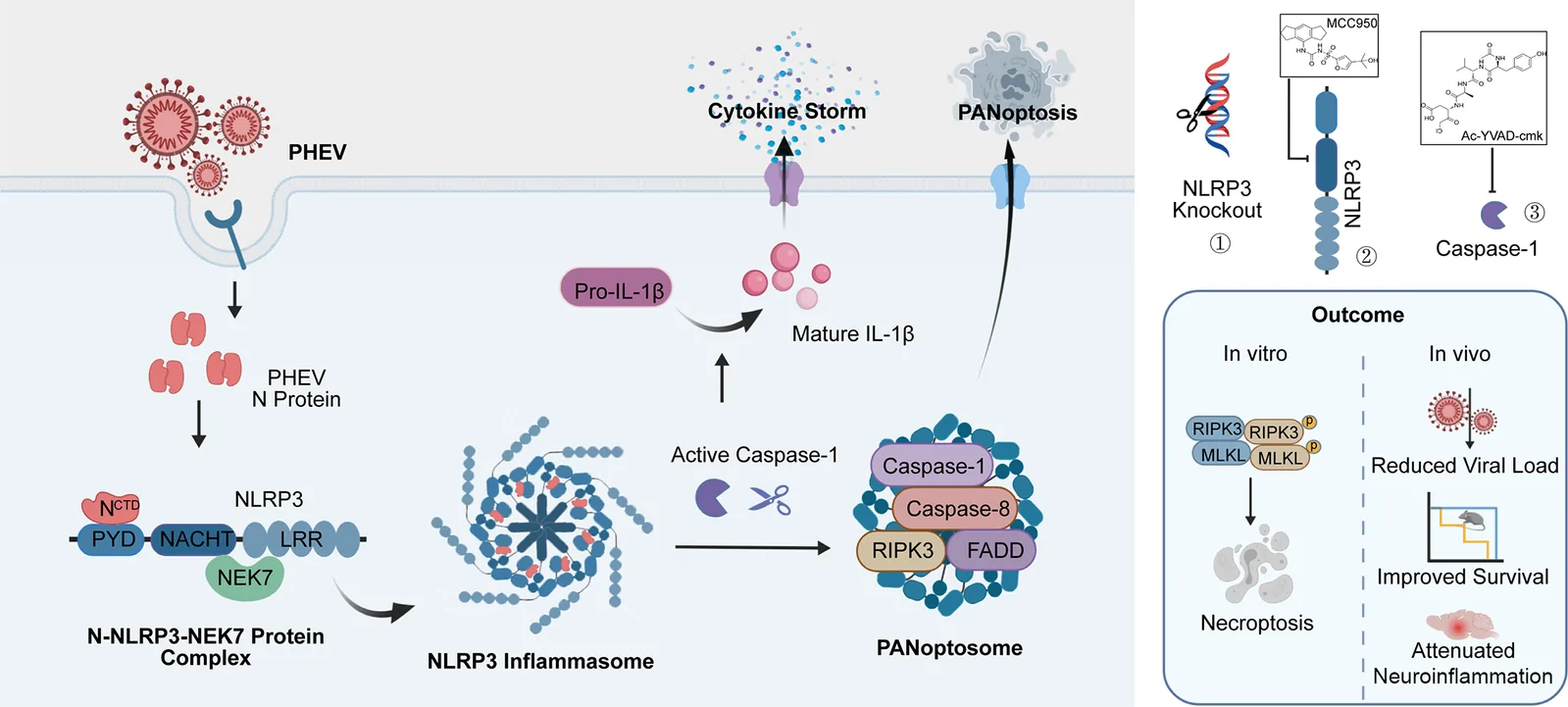
Working model of PHEV N protein activates the NLRP3 inflammasome. The C-terminal domain (CTD) of the PHEV nucleocapsid (N) protein binds the pyrin domain (PYD) of NLRP3, triggering inflammasome assembly and caspase-1 activation. This leads to mitochondrial dysfunction, characterized by ROS overproduction and loss of ΔΨm. These events converge to nucleate a PANoptosome, which coordinately activates pyroptosis, apoptosis, and necroptosis (PANoptosis), ultimately resulting in neuronal death and tissue damage in the central nervous system.

In summary, these data demonstrate that PHEV infection triggers severe mitochondrial dysfunction, characterized by ROS overproduction and loss of membrane potential. This damage converges on the NLRP3 inflammasome to nucleate the assembly of a PANoptosome, promoting the coordinated activation of PANoptosis (Fig. 8). Genetic ablation of NLRP3 not only dampens this integrated death cascade and alters its modality but also underscores the inflammasome’s central role in amplifying virus-induced cytopathology.

## DISCUSSION

Viral encephalitis remains a major global health burden, where dysregulated neuroinflammation is a central driver of pathology. Neurotropic coronaviruses, such as SARS-CoV-2, HCoV-OC43, MHV, and PHEV, illustrate the severe neurological consequences of such infections (1–3). PHEV circulates worldwide via subclinical infection in swine herds, posing a significant threat to naive neonatal piglets. The virus displays marked neurotropism, spreading via peripheral nerves (e.g., trigeminal and vagus) to the CNS and causing encephalomyelitis (12). In piglets under 4 weeks of age, infection leads to severe vomiting and wasting disease (VWD), influenza-like illness (ILI), and often fatal neurological dysfunction (41, 42). Using the neurotropic porcine betacoronavirus PHEV as a model, this study identifies a mechanism by which the viral N protein promotes NLRP3 inflammasome activation and contributes to neuropathogenesis. Our analysis provides insight into coronavirus-host innate immune interactions and supports a pathogenic role for NLRP3 inflammasome signaling during PHEV infection.

Although inflammasome signaling is often considered part of antiviral innate immunity, its role is increasingly recognized as context-dependent. In highly inflammatory viral infections, particularly in coronavirus infections, excessive or sustained NLRP3 activation may become pathogenic by amplifying IL-1β-driven inflammation, promoting tissue damage, and establishing feedforward inflammatory circuits that favor disease progression (43–45). Our data support such a model in PHEV infection. The consistent reduction in the CNS viral burden, microglia activation, and production of neuroinflammatory factors following pharmacological or genetic disruption of NLRP3 signaling suggests that this pathway contributes to a tissue environment that favors PHEV propagation and immunopathology, revealing a host-pathogen interface relevant to disease progression. At present, the basis for this proviral association remains unresolved. We cannot exclude, however, that NLRP3 deficiency also alters cellular permissiveness or preserves other antiviral pathways, such as interferon responses (46), that may become compromised during excessive inflammasome activation. Therefore, while our data identify NLRP3 as a critical pathogenic host factor during PHEV infection, the precise mechanism by which it enhances viral propagation warrants further investigation.

NLRP3 activation has been reported during infection with several coronaviruses, including SARS-CoV-1, SARS-CoV-2, and MHV, but the viral and host determinants that control this response are not fully defined. For example, SARS-CoV-1 RNA triggers NLRP3 via TLR8, and its spike (S) glycoprotein may modulate the inflammasome through ion channel activity or host miRNA regulation (47–49). Although the SARS-CoV-2 N protein has been shown to bind NLRP3, and its spike S1 subunit can increase NLRP3 expression in microglial cells, the specific interacting domains and functional outcomes in the nervous system remain unclear (10, 50). Likewise, while MHV activates NLRP3 and drives pathologies such as pneumonia or complex cell death, the viral factor that directly engages NLRP3 has not been identified (51, 52). Here, we provide biochemical and imaging evidence that the PHEV N protein, through its C-terminal domain (CTD), binds to the NLRP3 pyrin domain (PYD). Unlike the lyssavirus M protein that competitively blocks NLRP3-NEK7 binding (53), PHEV N engages NLRP3 without disrupting the essential NLRP3-NEK7 interaction, a critical checkpoint for inflammasome activation (54, 55). This CTD-PYD association may facilitate NLRP3-ASC complex formation and promote inflammasome assembly. In addition to PHEV, we found that the CTD of SARS-CoV-2 N also binds the PYD domain of NLRP3, extending our observations beyond a single virus. This result, together with previous reports that SARS-CoV-2 N associates with NLRP3 (10), suggests that direct PYD engagement by N-CTD may constitute a conserved coronaviral mechanism for inflammasome activation. However, whether this shared interaction leads to equivalent downstream inflammatory outcomes in human cells remains to be determined. Interestingly, this notion is reinforced by recent work, which developed a broad-spectrum aptamer (NApt8-3) that blocks the N-protein-NLRP3 interactions across diverse coronaviruses, including SARS-CoV-2, SARS-CoV, MERS-CoV, and endemic HCoV-OC43 and HCoV-229E (56). This expanded observation strengthens the broader relevance of our study, particularly in the context of coronavirus-associated inflammatory and neurological disease.

In addition to the N protein, we observed that PHEV E also enhanced IL-1β release (Fig. 3A), which is notable in light of prior work on coronavirus E proteins. SARS-CoV E has been reported to promote NLRP3 activation indirectly through viroporin-dependent disruption of Ca²^+^ homeostasis, rather than through direct binding to inflammasome core components (35). Consistent with this model, PHEV E promoted ASC complex formation but did not detectably associate with NLRP3 in our assay, suggesting that PHEV E and N may stimulate inflammasome signaling through mechanistically distinct routes. Thus, we suggest that the PHEV N protein functions as a viral factor that engages the NLRP3 PYD, identifying a virus-host interface that may be explored for host-directed therapeutic intervention. Given the inherent difficulty of mutating the N protein in the infectious virus without causing pleiotropic effects, the precise *in vivo* relevance of the N–NLRP3 interaction remains to be further investigated using alternative approaches, such as conditional expression systems or complementation strategies.

Beyond its role in neuropathology, NLRP3 activation orchestrated a profound shift in cell fate and can be affected by indirect signals such as dysregulated ion flux, mitochondrial ROS production, and lysosomal destabilization (57–59). In PHEV infection, this shift is accompanied by extensive mitochondrial dysfunction, characterized by ROS overproduction and loss of membrane potential, ultimately culminating in PANoptosis (60). When NLRP3 was defective, these mitochondrial alterations were markedly attenuated, and the dominant cell death pathway shifted from PANoptosis to a necroptosis-like profile—a phenomenon also reported during hepatitis E virus infection (61). The ability of PHEV to provoke NLRP3-dependent PANoptosis suggests a mechanism by which the virus amplifies cytopathology, potentially facilitating viral release, tissue damage, and immune clearance. To further explore the relationship between mitochondrial stress and NLRP3 activation, we pretreated infected cells with the mitochondrial ROS scavenger MitoTEMPO, which partially reduced ROS accumulation (Fig. S3I). Although these data do not definitively resolve the precise directionality between mitochondrial ROS and NLRP3 engagement, they support a model in which mitochondrial dysfunction and NLRP3 signaling are interconnected through a feed-forward circuit that amplifies inflammatory injury during later stages of infection. Thus, modulating these cell death pathways via targeting the NLRP3 inflammasomes or specific suborganelles represents a promising therapeutic strategy (55).

In conclusion, this study identifies a mechanism by which the neurotropic coronavirus PHEV engages host innate immune signaling. The viral N protein associates with the NLRP3 PYD and promotes inflammasome assembly, increased viral burden, and PANoptosis-associated cell-death signaling. Whether similar N-NLRP3 interactions operate in other coronaviruses and contribute to neurological disease warrants further investigation. It is a point particularly relevant to SARS-CoV-2, wherein NLRP3 activation is linked to neurological complications and disease severity. Together, these findings support further exploration of NLRP3 inflammasome signaling and viral N protein-host interactions as candidate targets for mitigating coronavirus-associated neuroinflammation.

## MATERIALS AND METHODS

### Cells and virus

Embryonic kidney cell lines (HEK293T), mouse monocytic cell lines (RAW264.7), mouse neuroblastoma cell lines (N2a), and porcine alveolar macrophage cell lines (PAM) were preserved in our laboratory. N2a cells were cultured in DMEM (6% fetal bovine serum), HEK293T cells were cultured in DMEM (8% fetal bovine serum), and RAW264.7 and PAM cells were cultured in DMEM (10% fetal bovine serum) (Gibco). All the medium was supplemented with 100 U/mL penicillin and 100 μg/mL streptomycin and maintained at 37°C in a 5% CO_2_ incubator. PHEV (GenBank: MF083115.1) was used and propagated by passage in N2a cells or RAW264.7 cells. For the infection assay, the cells were infected with PHEV at a multiplicity of infection (MOI) of 1 for 0–36 h. For positive controls of NLRP3 inflammasome activation, the cells were primed with LPS (1 μg/mL) for 6 h followed by ATP stimulation (2.5 mM) for 30 min. For pharmacological inhibitor assays, the cells were treated with inhibitors (MCC950, Ac-YVAD-cmk, or BAY 11-7082) either pre-treatment (1 h before infection), co-treatment (simultaneously with infection), or post-treatment (1 h after infection). Supernatants were collected for measurement of IL-6, IL-18, IL-1β, and TNF-α proteins.

### Mice

C57BL/6 *Nlrp3^−/−^* mice were provided by Dr. Yu from the State Key Laboratory of Reproductive Regulation and Breeding of Grassland Livestock, College of Life Sciences, Inner Mongolia University, Hohhot, China. Wild-type C57BL/6 mice were purchased from the Laboratory Animal Center of Jilin University, Changchun, China. Mice were bred and maintained under specific pathogen-free conditions. Four-week-old Wt or *Nlrp3^−/−^* C57BL/6 mice (male) were randomly allocated and intranasally inoculated with 30 µL PHEV (10^6.8^ TCID_50_/0.1 mL). For *in vivo* treatment, MCC950 (Selleck, CP-456773) and Ac-YVAD-cmk (Sigma-Aldrich, SML0429) were administered to mice by intraperitoneal injection at doses of 50 mg/kg (in H₂O) and 0.1 mg/kg (in DMSO), respectively. Subsequently, PHEV-infected mice at 2 dpi, 4 dpi, or 6 dpi were sacrificed by CO_2_ asphyxiation according to animal handling guidelines. After sacrifice, the brain of each mouse was surgically excised and placed on an ice pad for further testing.

### Quantitative real-time polymerase PCR (qRT-PCR)

The total RNA was extracted using RNAiso Plus (Takara, 9109) according to the manufacturer’s instructions; the RNA was then reverse-transcribed into cDNA at 42°C for 1 h followed by 72°C for 10 min with reverse transcriptase. Real-time qRT-PCR was performed using the 2× SYBR Green Master Mix (Bimake, B21202). The relative mRNA expression was normalized to the housekeeping genes GAPDH or β-actin using the 2^–ΔΔCt^ method, and fold changes were calculated relative to specified control groups. The real-time PCR primers (Table S1) were designed by Primer-blast, NCBI (https://www.ncbi.nlm.nih.gov/).

### Western blotting

The cells used in the experiment were based on the experimental requirements and were infected with PHEV or treated with drugs at the indicated times of infection. The cells were lysed in RIPA buffer (1% Triton X-100 and 1 mM phenylmethylsulfonyl fluoride in PBS). After centrifugation at 12,000 rpm, the supernatant of cell lysates (200 µL) was taken, resuspended in 5× SDS-PAGE loading buffer (CWbio, CW0027S), and boiled. The samples were fractionated on 10% SDS-PAGE gels and subsequently transferred to polyvinylidene difluoride (PVDF; MCE, IPVH00010) membranes. The membranes were blocked and treated with a primary antibody and a secondary antibody (HRP). The membranes were ultimately visualized with an enhanced chemiluminescence (ECL) kit from Biosharp and then subjected to immunoblot analysis and quantified using ImageJ software. The antibodies used were as follows: mouse anti-PHEV antibody (stored in our lab), anti-NLRP3 antibody (Abcam, ab263899), rabbit anti-ASC antibody (Abcam, ab283684), rabbit anti-caspase-1 antibody (Abcam, ab179515), rabbit anti-IL-1β antibody (Abcam, ab234437), rabbit anti-p65(phospho) antibody (Abcam, ab76302), rabbit anti-IL-1β antibody (Abcam, ab234437), rabbit anti-caspase-3 antibody (CST, 9661), rabbit anti-caspase-7 antibody (Proteintech, 27155), rabbit anti-caspase-8 antibody (Proteintech, 13423), rabbit anti-GSDMD antibody (Abcam, ab209845), rabbit anti-GSDME antibody (Abcam, ab222408), rabbit anti-RIPK3 antibody (Proteintech, 29080), rabbit anti-RIPK1 antibody (Proteintech, 29932), mouse anti-MLKL antibody (Proteintech, 66675), rabbit anti-FADD antibody (Proteintech, 14906), rabbit anti-DRP1 antibody (Proteintech, 12957), and HRP-conjugated secondary antibodies (Proteintech).

### Co-immunoprecipitation assay

HEK293T or RAW264.7 cells grown to 70%–80% conﬂuence in 6-cm dishes were co-transfected with the indicated plasmids for 24–48 h. Transfected HEK293T cells were lysed in lysis buffer (500 μL Cell Complete Lysis Buffer for western and IP; Beyotime, P0037). The lysates were lysed on ice for 30 min and centrifuged at 12,000 rpm for 10 min to remove the debris. An aliquot of supernatants was used as input, and the rest of the supernatants were incubated with the indicated antibodies overnight at 4°C and mixed with rProtein A/G Beads 4FF (Smart-Lifesciences, SA032025) for 30 min at 25°C or 4 h at 4°C. Immunoprecipitates were washed three times with the respective lysis buffer, boiled in protein loading buffer, and analyzed by western blotting.

### Molecular docking

The UniProt code (UniProt ID: 7vtq) acquired from the Protein Data Bank pertains to the crystal structure of NLRP3. Pathogen proteins (PHEV-N) were modeled using SWISS-MODEL homology. The preliminary configuration of the simulation complex of NLRP3-N was acquired via GRAMM software for conventional docking protocols.

### Confocal microscopy

HEK293T or RAW264.7 cells grown overnight on the 20-mm glass slide were transferred into the 12-well culture plate. The cells treated according to experimental requirements were fixed in 4% paraformaldehyde and cold methanol, permeabilized in 0.5% Triton X-100, and blocked in 5% BSA. The cells were then incubated with primary antibodies, including rat anti-NLRP3 antibody (Invitrogen, MA5-23919), rabbit anti-ASC antibody (Abcam, ab283684), rabbit anti-GFP-tag antibody (Proteintech, 50430), mouse anti-His-tag antibody (Proteintech, 66005), rabbit anti-HA-tag antibody (Proteintech, 51064), rabbit anti-DYKDDDDK-tag antibody (Proteintech, 20543), and mouse anti-PHEV-N antibody (stored in our lab). The slides were labeled with the indicated fluorescent secondary antibodies, including Alexa Fluor 488-conjugated goat anti-rabbit antibody (CST, 4412), Alexa Fluor 488-conjugated goat anti-mouse antibody (CST, 4408), Alexa Fluor 594-conjugated goat anti-rabbit antibody (CST, 8889), Alexa Fluor 594-conjugated goat anti-mouse antibody (CST, 8890), Alexa Fluor 647-conjugated goat anti-rabbit antibody (CST, 4414), and Alexa Fluor 647-conjugated goat anti-rat antibody (CST, 4418) for 1 h at 37℃ and then incubated with 4,6-diamidino-2-phenylindole (DAPI, Beyotime, C1005). Images were taken using the Olympus FV3000 confocal microscope (Olympus) and sealed with Antifade Mounting Medium with Hoechst (Beyotime) and viewed by a laser scanning confocal fluorescence microscope (Olympus FluoView FV1000). All images were analyzed using ImageJ.

### GST pull-down assay

Recombinant GST-tagged PHEV N protein and GST alone were expressed in *E. coli* BL21(DE3) and purified using glutathione Sepharose 4B beads (GE Healthcare) according to the manufacturer’s instructions. Cell lysates containing NLRP3 were incubated with equal amounts of immobilized GST or GST-N beads in binding buffer (50 mM Tris-HCl [pH 7.4], 150 mM NaCl, 0.5% NP-40, 1 mM DTT) at 4°C overnight. After extensive washing with buffer containing 300 mM NaCl, bound proteins were eluted by boiling in SDS sample buffer, resolved by SDS-PAGE, and analyzed by western blotting using anti-NLRP3 and anti-GST antibodies.

### ASC oligomerization analysis

RAW264.7 cells stimulated with PHEV were lysed in lysis buffer, gently shaken at 4°C for 30 min, and centrifuged at 6,000 × *g* at 4°C for 15 min. The supernatant was used to detect ASC (P25). The pellets were washed three times with TBS and re-suspended in 200 μL TBS. DSS (2 mM) was added to the re-suspended pellets, which were cross-linked at 37°C for 30 min. The samples were centrifuged at 6,000 × *g* for 10 min; the cross-linked pellets were re-suspended in 40 μL 1× SDS loading buffer, boiled for 10 min, and analyzed by western blotting.

### Mitochondrial membrane potential assay

After treating of the cells as described above, the old medium was aspirated. The cells were loaded with JC-1 working solution and maintained in a cell incubator at 37°C for 20 min in the dark. The supernatant was aspirated, and cells were washed with JC-1 staining buffer twice. The mitochondrial membrane potential is high in healthy cells, and JC-1 exists as aggregates with bright red fluorescence. When the mitochondrial membrane potential decreased, the number of JC-1 monomers increased and showed green fluorescence. Then, the cell fluorescence images were observed and captured using a laser scanning microscope.

### Reactive oxygen species (ROS) detection

DCFH-DA is a fluorescent probe used to detect reactive oxygen species (ROS). When the fluorescent probe is added to the treated cells, DCFH-DA itself does not have fluorescence but can freely penetrate the cell membrane. ROS inside the cell can oxidize non-fluorescent DCFH to generate fluorescent DCF, and the fluorescence intensity is proportional to the ROS level. Wild-type and NLRP3-knockout RAW264.7 macrophages were infected with PHEV for 24 h, followed by fluorescence intensity quantification. To evaluate the mitochondrial ROS involvement, the cells were pretreated with 200 μM mitoTEMPO (MCE, HY-112879) for 24 h prior to PHEV infection. Untreated Wt cells and Rosup-treated cells served as the negative and positive controls, respectively. Fluorescence signals are detected at a maximum excitation wavelength of 480 nm and a maximum emission wavelength of 525 nm using equipment such as fluorescence microscopy, flow cytometry, or laser confocal microscopy.

### Statistical analyses

Data were analyzed using SPSS version 19.0, and diagrams were generated with GraphPad Prism 8.0. The data are presented as the mean ± standard deviation (SD). *, **, and *** represent *P* < 0.05, 0.01, and 0.001, respectively.

## Data Availability

The data that support the findings of this study are available from the corresponding author upon reasonable request.

## SUPPLEMENTAL MATERIAL

Supplemental material mbio.01725-26-s0001.docx Fig. S1 to S3; Table S1.

ASM does not own the copyrights to Supplemental Material that may be linked to, or accessed through, an article. The authors have granted ASM a non-exclusive, world-wide license to publish the Supplemental Material files. Please contact the corresponding author directly for reuse.
