# Supplementary material for: Porcine coronavirus promotes NLRP3 inflammasome activation during virus-induced neuroinflammation
Source: mBio. 2026 Aug 17;17(9):e01725-26. doi: 10.1128/mbio.01725-26 (PMC13556248; doi:10.1128/mbio.01725-26)
Supplement: Supplementary file 1 [file mbio.01725-26-s0001.docx]

**Figure S1. PHEV infection triggers inflammation.**

(A) Confocal microscopy of PAMs infected with PHEV for 24 h, stained for PHEV-N (green) and nuclei were counterstained with Hoechst (blue). Scale bars, 40 μm. (B) PHEV and indicated genes mRNA expression in PHEV-infected PAMs. (C) PHEV and indicated genes mRNA expression in PHEV-infected BV2 macrophages. (D) Viral titer in RAW264.7 cells infected with PHEV for 6h, 12h, 24h, 36h. (E) Confocal microscopy of RAW264.7 cells infected with PHEV for 6h, 24h, 36h, stained for PHEV-N (green) and nuclei were counterstained with Hoechst (blue). Scale bars, 10 μm. (F) Activation of NF‑κB signaling (phospho‑p65) in RAW264.7 cells infected with PHEV (0–36 h) or stimulated with LPS+ATP, assessed by immunoblotting. (G) Cytotoxicity of the NF‑κB inhibitor BAY 11‑7082 in RAW264.7 cells, determined by CCK‑8 assay after 24  h. Data are mean ± SD (n = 3); *P < 0.05, **P < 0.01 (unpaired two-tailed t-test).

**Figure S2. Coronavirus-NLRP3 Protein Interaction.**

(A) Viral N protein levels in N2a cells infected with PHEV for 24  h. (B) HEK293T cells were transfected with EGFP-E, HIS-ASC, and either MYC-NLRP3. Confocal images show zoom (right). Nuclei are shown in cyan. Scale bars, 10 µm. (C) Co‑IP in HEK293T cells co‑expressing MYC‑NLRP3 with HIS-ASC, GFP-E, or GFP. Cell lysates were immunoprecipitated using anti-HIS antibody, and analyzed using anti-HIS and anti-MYC antibodies. (D) Co‑IP in HEK293T cells co‑expressing MYC‑NLRP3 with full‑length GFP-E or GFP. Cell lysates were immunoprecipitated using anti-GFP antibody, and analyzed using anti-GFP and anti-MYC antibodies. (E) Co‑IP in HEK293T cells co‑expressing MYC‑NLRP3-PYD with full‑length HIS-SARS2-N-CTD or HIS vector. Cell lysates were immunoprecipitated using anti-HIS antibody, and analyzed using anti-HIS and anti-MYC antibodies.

**Figure S3. NLRP3 inflammasome activation promotes PHEV replication and neuroinflammation via enhanced oxidative stress.**

(A, B) Viability of RAW264.7 cells treated with the NLRP3 inhibitor MCC950 or the CASP1 inhibitor Ac‑YVAD‑cmk for 24  h by CCK‑8 assay. (C) Viral titer in brain homogenates following pharmacological inhibition. C57BL/6 mice (n=6 per group) were intranasally infected with PHEV and treated intraperitoneally with vehicle, MCC950 (50 mg/kg), or Ac‑YVAD‑cmk (0.1 mg/kg). Viral titers in brain homogenates at 6 dpi were determined by TCID₅₀ assay and are expressed as log₁₀TCID₅₀/g tissue. (D) Viral titer in brain homogenates of NLRP3-deficient mice. Wild-type (Wt) and *Nlrp3⁻^/^⁻* C57BL/6 mice (n=6 per group) were intranasally infected with PHEV. Viral titers in brain homogenates at 6 dpi were determined by TCID₅₀ assay (log₁₀TCID₅₀/g tissue). (E) Immunohistochemical images of brain sections from the mice at 6 dpi. Sections were stained for the microglial marker IBA1 (Scale bars, 50 µm) and the astrocytic marker GFAP (Scale bars, 100 µm). Images are representative of three independent experiments with consistent observations. (F) Quantitative analysis of IBA1^+^ microglia and GFAP^+^ astrocytes in brain sections, corresponding to the representative images shown in (E). (G) Immunohistochemical images of brain sections from the Wt and *Nlrp3⁻^/^⁻* mice at 6 dpi, stained for IL-1β. (H) ROS production detected by DCFH‑DA quantified in PHEV-infected RAW264.7 macrophages. Rosup group served as a positive control. (I) RAW264.7 macrophages were pretreated with 200 μM mitoTEMPO for 24 h prior to PHEV infection. Untreated cells (NC) and Rosup-treated cells served as the negative and positive controls, respectively. Data are represented as the means ± SD, n = 3; * P < 0.05, **P < 0.01, ***P < 0.001 (unpaired two-tailed t-test).

**Table S1. Sequences of all molecules used for qRT-PCR assays.**

| **Gene name** | **Sequence (5’-3’)** |
| --- | --- |
| NLRP1-F | GCTTTGGTGCCAGACTTCCT |
| NLRP1-R | TGATGTGTTCGTCCCCAGGC |
| IL-18-F | AGCTCTTCTACCAGCAAACATC |
| IL-18-R | CTTCCAACTGAGAGGCTGTGC |
| NLRP10-F | TCAAGACGCTGAAGTTCCACT |
| NLRP10-R | TGCTCCGTACATTGAAATCAGTT |
| NLRP6-F | TGGTGAGTGGAAAGCAGCTA |
| NLRP6-R | CCTTCCTCCACTGCTTCTGA |
| NLRC4-F | CCTGTTGAAACATTTGGAGGAG |
| NLRC4-R | CTGACTAATGCTGGATCAGGTAGA |
| AIM2-F | GCCCAGCAGGAATCTATCAGAGAAG |
| AIM2-R | AGCCACTGTAGCATGAAACATCTCC |
| IFI16-F | GAAGTGCCAGCGTAACTCCTAA |
| IFI16-R | TGATTGTGGTCAGTCGTCCAT |
| GAPDH-F | 5'-AGGTCGGTGTGAACGGATTTG-3' |
| GAPDH-R | 5'-TGTAGACCATGTAGTTGAGGTCA-3' |
| NLRP3-F | 5'-TGGAGACACAGGACTCAGGC-3' |
| NLRP3-R | 5'-CATTTCACCCAACTGTAGGC-3' |
| Caspase-1-F | TGCCGTGGAGAGAAACAA |
| Caspase-1-R | ATGAAAAGTGAGCCCCTG |
| PHEV-F | TCTGGGAATCCTGACGAG |
| PHEV-R | AGGCGCTGCAACACTTAC |
| ASC-F | GAAGCTGCTGACAGTGCAAC |
| ASC-R | CTCTGCTCCAGGTCCATCAC |
| IL-6-F | GGCGGATCGGATGTTGTGAT |
| IL-6-R | GGACCCCAGACAATCGGTTG |
| TNF-α-F | GGAACACGTCGTGGGATAATG |
| TNF-α-R | GGCAGACTTTGGATGCTTCTT |
| IL-18-F | CCTACTTCAGCATCCTCTACTGG |
| IL-18-R | AGGGTTTCTTGAGAAGGGGAC |
| IL-1β-F | GCAACTGTTCCTGAACTCAACT |
| IL-1β-R | ATCTTTTGGGGTCCGTCAACT |
